# A novel multiplex assay combining autoantibodies plus PSA has potential implications for classification of prostate cancer from non-malignant cases

**DOI:** 10.1186/1479-5876-9-43

**Published:** 2011-04-19

**Authors:** Chong Xie, Hyun J Kim, Jonathan G Haw, Anusha Kalbasi, Brian K Gardner, Gang Li, Jianyu Rao, David Chia, Monty Liong, Rubio R Punzalan, Leonard S Marks, Allan J Pantuck, Alexandre de la Taille, Guomin Wang, Hideki Mukouyama, Gang Zeng

**Affiliations:** 1 Department of Urology, Zhongshan Hospital of Fudan University, No.180 Fenglin Road, Shanghai 200032, China; 2 Department of Radiology, David Geffen School of Medicine at UCLA, 10833 Le Conte Ave, Los Angeles, CA 90095-1721, USA; 3 Department of Urology, David Geffen School of Medicine at UCLA, 10833 Le Conte Ave, Los Angeles, CA 90095-1738, USA; 4 Department of Medicine, David Geffen School of Medicine at UCLA, 10833 Le Conte Ave, Los Angeles, CA 90095-1732, USA; 5 Department of Biostatistics, UCLA School of Public Health, 10833 Le Conte Ave, Los Angeles, CA 90095-1772, USA; 6 Department of Pathology and Laboratory Medicine, David Geffen School of Medicine at UCLA, 10833 Le Conte Ave, Los Angeles, CA 90095-1738, USA; 7 Department of Chemistry and Biochemistry, 607 Charles E. Young Drive East, Los Angeles, CA 90095-1569, USA; 8 Advanced Medical Analysis, LLC, 1941 Walker Ave, Monrovia, CA 91016, USA; 9 Department of Urology, CHU Henri Mondor, Créteil U955 E907, France; 10 Department of Urology, Okinawa Nambu Tokushukai Hospital, 80 Hokama, Yaese-cho, Shimajiri-gun, Okinawa 901-0417, Japan

## Abstract

**Background:**

The lack of sufficient specificity and sensitivity among conventional cancer biomarkers, such as prostate specific antigen (PSA) for prostate cancer has been widely recognized after several decades of clinical implications. Autoantibodies (autoAb) among others are being extensively investigated as potential substitute markers, but remain elusive. One major obstacle is the lack of a sensitive and multiplex approach for quantifying autoAb against a large panel of clinically relevant tumor-associated antigens (TAA).

**Methods:**

To circumvent preparation of phage lysates and purification of recombinant proteins, we identified B cell epitopes from a number of previously defined prostate cancer-associated antigens (PCAA). Peptide epitopes from cancer/testis antigen NY-ESO-1, XAGE-1b, SSX-2,4, as well as prostate cancer overexpressed antigen AMACR, p90 autoantigen, and LEDGF were then conjugated with seroMAP microspheres to allow multiplex measurement of autoAb present in serum samples. Moreover, simultaneous quantification of autoAb plus total PSA was achieved in one reaction, and termed the "A+PSA" assay.

**Results:**

Peptide epitopes from the above 6 PCAA were identified and confirmed that autoAb against these peptide epitopes reacted specifically with the full-length protein. A pilot study was conducted with the A+PSA assay using pre-surgery sera from 131 biopsy-confirmed prostate cancer patients and 121 benign prostatic hyperplasia and/or prostatitis patients. A logistic regression-based A+PSA index was found to enhance sensitivities and specificities over PSA alone in distinguishing prostate cancer from nonmalignant cases. The A+PSA index also reduced false positive rate and improved the area under a receiver operating characteristic curve.

**Conclusions:**

The A+PSA assay represents a novel platform that integrates autoAb signatures with a conventional cancer biomarker, which may aid in the diagnosis and prognosis of prostate cancer and others.

## Background

Both the cellular and humoral arms of the human immune system recognize tumor-associated antigens (TAA) derived from endogenously arising cancer cells. Of particular interest to the serological analysis of human cancers is a panel of clinically relevant TAA recognized by autoAb present in the serum of cancer patients including those with prostate cancers [1,2]. In prostate cancer, autoAb-recognized prostate cancer-associated antigens (PCAA) may be divided into two categories: 1) autoAb recognize α-methylacyl-CoA (AMACR) [3,4], p90 autoantigen [5], and lens epithelium-derived growth factor p75 (LEDGF) [6], which have low levels of expression in normal tissues, but are overexpressed in prostate cancer; 2) autoAb react against cancer/testis antigens such as NY-ESO-1 [7], SSX-2,4 [8], and XAGE-1b [9], which are observed only in cancer patients but not healthy donors (HD) or patients with benign conditions. Cancer/testis antigens are by far the most cancer-specific TAA, which are shared by a number of solid tumors including prostate cancer, lung cancer, and so on. In normal tissues, they are only expressed in immune-privileged germline cells. In this study, we focused on a panel of clinically relevant PCAA, whose expression in prostate cancer tissues and autoAb presence in serum samples have been verified by multiple groups. AutoAb against these targets are also observed prominently in prostate cancer patients than healthy donors.

In contrast to conventional biomarkers produced by tumor cells such as PSA, autoAb against clinically relevant TAA are produced by the body in response to neoplastic transformation. Spontaneous autoAb present in patients' serum samples may reflect cancer-related inflammation, immunocompetence of the host, and immunogenicity of the endogenously arising cancer [10,11]. Even though more and more studies have shown the significance of circulating autoAb in serving cancer detection, diagnosis, prognosis, and other areas [12-14], sensitive and cost-effective detection of autoAb against multiple TAA still lacks that may serve for clinical laboratories.

Currently, two main strategies are used for broad-based profiling of circulating autoAb: serological surveys using phage lysates encoding specific TAA [4], protein array and ELISA-based approaches using purified recombinant proteins [15-17]. The former approach requires large amounts of sera individually pre-adsorbed with *E. coli *phage lysates for reduction of background; the latter require the purification of proteins encoding individual TAA. To circumvent the requirement of purifying phage lysates or individual TAA protein, we have focused on targeted identification of B cell epitopes from TAA [18], and developed a novel multiplex assay platform that quantifies autoAb plus total PSA in a single reaction for prostate cancer.

## Methods

### Prediction, screening and validation of B-cell epitopes from PCAA

The study focused on 6 PCAA, namely NY-ESO-1, SSX-2,4, XAGE-1b, AMACR, p90, and LEDGF. All had been reported by multiple groups with data on gene expression and autoAb presence in prostate cancer patients. As previously described [18], prediction and screening of peptide epitopes was conducted using classic ELISA. Peptides were considered positive based on recognition by serum samples from prostate cancer patients (n > 50) but not healthy donors (n > 20). Then, peptide-reacting serum samples were verified for recognition of the full-length or a truncated recombinant protein using Western blot. Only after such a procedure, a validated peptide was conjugated onto seroMAP microbeads for multiplex measurement. All peptides involved in this study were synthesized at Genscript Inc. (Piscataway, NJ) and GeneMedicine, Inc. (San Antonio, TX). Historical serum samples from cancer and HD as described previously [18] were used for identification of peptide epitopes, which were independent of those used in the subsequent study comparing A+PSA index with PSA. In the case of identifying peptide epitopes from shared cancer/testis antigen XAGE-1b and SSX2,4, serum samples from NSCLC were used. This choice was made based on higher frequency of seropositive subjects in NSCLC and the fact that peptides from shared antigens identified using one type of cancer patients can be equally well recognized by prostate cancer patients [18].

### Clinical and demographic characteristics of serum donors involved in the study

All serum samples were collected under institutional review board-approved protocols from UCLA (IRB#06-03-044) and collaborating hospitals, and stored at -20°C until use. Serum samples from normal healthy subjects were collected at the time of blood donation in subjects routinely screened to exclude the presence of concomitant disease such as cancer according to standard blood bank policies. Serum samples from biopsy-confirmed prostate cancer patients were collected at the time of biopsy and prior to surgery. Patients with BPH and/or prostatitis, specified as non-cancer or BPH/prostatitis patients throughout this manuscript, were those with clinical signs and symptoms, for instance, characteristic lower urinary tract symptoms, International Prostate Symptom Scores, urinary leukocytes, and so on. These patients were subsequently underwent a routine fine needle prostate biopsy with at least 6-12 samples taken showing no evidence of prostate cancer. Table 1 shows the demographic and clinical characteristics of the subjects involved in the comparison of A+PSA with PSA alone. Additional file 1 illustrates the distribution of their total PSA values, which were measured using a standard ELISA approach according to the manufacturer's recommendations at the time of diagnosis.

**Table 1:** Demographic and clinical characteristics of patients involved in the study

Subjects	HD	BPH/Prostatitis	Prostate Cancer
	n = 124	n = 121	n = 131
Age (year)			
unknown	124	2	4
<40		2	1
40-49		4	2
50-59		16	2
60-69		30	14
70-79		46	38
>80		21	20

Collection site*			
Japan	84	121	81
U.S.	40	0	50

Gleason Scores			
unknown			10
< and = 6			61
7			29
> and = 8			31

*Race is not known, samples are only classified based on the collection site. Note that this pilot study is focused on comparing A+PSA with the PSA assay, cohort size was not sufficient to match samples according to potential clinical co-founders.

Since this was a pilot study, cohort size and relevant parameters such as age, racial and ethnical background were not sufficient to match samples according to potential clinical co-founders. However, all samples themselves were handled and stored according to the same conditions prior to assay; and normalization with samples from HD was conducted when needed in order to minimize experiment-to-experiment variations.

### Conjugation of peptide epitopes with seroMAP mircrobeads and conduct of seroMAP-based assays

Conjugation of peptide epitopes defined in this study onto seroMAP beads was conducted according to the manufacturer's recommendations (Luminex Corporation, Austin, TX). In the final configuration of the A+PSA assay, seroMAP microbeads region 001 were conjugated with the NY-ESO-1 peptide epitope as previously reported [18], region 010 with the XAGE-1b epitope (amino acid 1-25), region 020 with the SSX2,4 epitope (amino acid 110-139), region 030 with the AMACR epitope (amino acid 251-281), region 040 with the p90 autoantigen epitope (amino acid 796-827), region 050 with a control peptide from β-galactosidase, and region 060 with the LEDGF epitope (amino acid 448-468). A 96-well filter bottom plate (Millipore, Billerica, MA) was pre-washed followed by addition of blocking buffer and incubation for 1 hour at room temperature. About 50 μl of serum samples pre-diluted at 1 to 10, 1 to 20, and 1 to 50 were mixed with an equal volume of the above-conjugated seroMAP microbeads at 5000 beads/region, and were added to each well. After one hour of incubation, plates were washed 3 times, followed by addition of 100 μl PE-labeled detection Ab (Ab against human IgG and Ab against human total PSA) to each well. After 30 min, plates were washed 3 times, and added 100 μl blocking buffer into each well. The plate was read by Bioplex-200 (Bio-Rad Laboratories, Hercules, CA) to obtain the mean florescent intensity (MFI) for each seroMAP region.

In addition to measuring autoAb, seroMAP microbeads region 100 were conjugated with a monoclonal Ab against human PSA (Biocon, Inc. Rockville, MD) to quantify total PSA levels. seroMAP-based PSA quantification was compared with standard ELISA-based PSA assays (American Qualex) and also made compatible with the measurement of the above-mentioned 6 autoAb to constitute the A+PSA assay.

### Comparison of signal to noise ratios of seroMAP- and ELISA-based approaches for autoAb measurement

AutoAb present in patients' serum samples were previously measured using a standardized ELISA approach [18]. In brief, 1 μg of a synthetic peptide was diluted in 5 ml phosphate buffered saline (PBS) and adsorbed onto a 96-well MaxiSorp plate (Nunc, Denmark) overnight at room temperature. Control plates were coated with bovine serum albumin (BSA) at 15 μg/plate or about 150 ng/well. Plates were blocked with 5% Fetal Bovine Serum in PBST (PBS plus 0.05% Tween-20) for at least 2 hours, washed with PBST, and loaded with 100 μl of serum samples diluted at 1:25, 1:125, and 1:625 with PBST containing 5% Fetal Bovine Serum. After a 2-hour incubation at room temperature, plates were washed, and loaded with secondary antibodies (goat anti-human immunoglobulin conjugated with horseradish peroxidase, Sigma Co., St. Louis, MO) diluted with 5% Fetal Bovine Serum in PBST. Plates were developed after a one-hour incubation, and absorbance at 450 nm was read by using an ELISA reader. Signal to noise ratio for ELISA-based approaches was defined as the OD against a target epitope/average OD from at least 8 HD. Signal to noise ratio for seroMAP-based approaches was defined as the specific MFI ratio against a target peptide/average specific MFI ratio against the same peptide from at least 8 HD, where the specific MFI ratio is defined as the MFI against a PCAA peptide/MFI against a control peptide.

### Statistical analysis and the logistic regression-based A+PSA index

To better predict prostate cancer, it is necessary to create an index integrating both autoAb against the 6 above-described PCAA and the patient's PSA status. For total PSA and autoAb against each peptide epitope, an index value was calculated based on the mean MFI ratio, which is defined as the florescent intensity against a specific peptide/florescent intensity against a control peptide. The Kolmogorov-Smirnov test was used in the 6 autoAb markers to determine if the histograms between prostate cancer and BPH and/or prostatitis differ significantly. The A+PSA index was defined as the probability of being prostate cancer, which was obtained by combining the six above referenced epitope indices with the PSA index using the logistic regression method

where each *Ni *represented the average MFI values for an autoAb from three dilutions, N_*PSA *_was the average MFI for PSA from three dilutions, and a0,...a7 were estimated regression coefficients of the logistic regression model. In the logistic regression model, the binary dependent variable is 1 for a patient with prostate cancer and 0 for a patient with nonmalignant conditions, for example, BPH and/or prostatitis. The receiver operating characteristic (ROC) curve was used to compare the diagnostic power between PSA alone and the combined A+PSA index for distinguishing prostate cancer from BPH and prostatitis in all subjects and subjects with 4-10 ng/ml PSA. Area Under the Curve (AUC) from Receiver operating characteristic (ROC) analysis was calculated from the logistic regression model. To avoid a potential overfitting issue in modeling and the testing within the same data set, the bootstrap method [19] was applied to construct 95% confidence intervals for the AUCs and test their difference. Values of P < 0.05 were considered statistically significant.

## Results

### Identification and validation of B cell epitopes from PCAA

**Figure 1:**
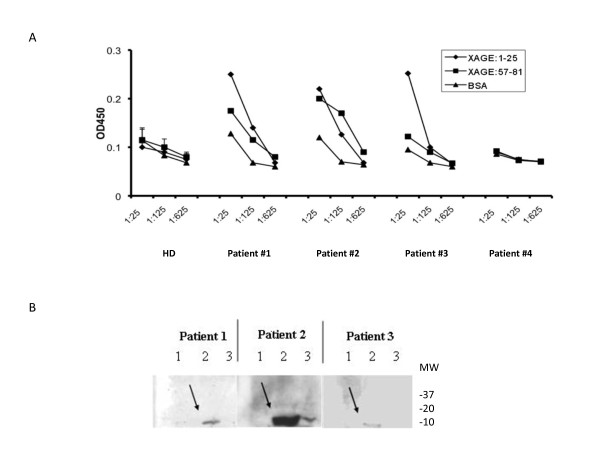
**Identification and validation of B cell epitopes from cancer/testis antigen XAGE-1b**. **(A)**. ELISA was used to screen candidate peptides from XAGE-1b for recognition by patients' sera. Three patients (#1-3) were positive for either XAGE-1b:1-25 or 57-81. Sera were diluted at 1:25, 1:125, and 1:625 with BSA serving as a control target. The mean OD of 8 HD and the OD of one seronegative patient (#4) are also shown. The use of sera from NSCLC patients for screening is due to higher frequency of Ab against these shared antigens in NSCLC patients. Previous work has shown that peptide epitopes identified using one type of sera are equally recognized by sera from other cancer patients. **(B)**. Western blots confirmed recognition of the full-length XAGE-1b protein. Lane 1, 2, and 3 contained, respectively, lysate from 293 cells transfected with a control plasmid, a plasmid encoding XAGE-1b (denoted with an arrow), and lysate from LNCaP-CL1 cells (expressing XAGE-1b but at a much lower level based on real-time PCR, data not shown).

Similar to NY-ESO-1, XAGE-1b and SSX-2,4 are cancer/testis antigens shared among cancers of the prostate, lung, breast and others [20,21]. To identify dominant B cell epitopes from XAGE-1b, computer-aided algorithms were applied to predict the peptide epitopes [18]. Two candidate peptides were screened by ELISA (Figure 1A) with serum samples from cancer patients. Three of 48 cancer patients were tested positive reacting with XAGE-1b peptides based on previously described criterion [18]. Two of the 3 seropositive patients reacted only with XAGE:1-25 peptide; while the other reacted with both XAGE:1-25 and XAGE:57-81 peptides. Western blot confirmed that sera recognizing the XAGE:1-25 peptide reacted with the full-length XAGE-1b protein from a transfected 293 cell line (Figure 1B).

Similarly, a SSX-2,4 peptide epitope was identified and confirmed with Western blot that the serum reacting with SSX-2,4:110-139 was able to recognize the full-length recombinant protein (Additional file 2). In addition, candidate peptides from AMACR, p90 autoantigen, and LEDGF were screened using serum samples from prostate cancer patients and control samples from HD (data not shown). Verification of the AMACR and LEDGF peptide epitopes by Western blot is also shown in Additional file 2.

### Peptide epitopes linked to seroMAP microspheres markedly improves signal-to-noise ratios over classic ELISA

**Figure 2:**
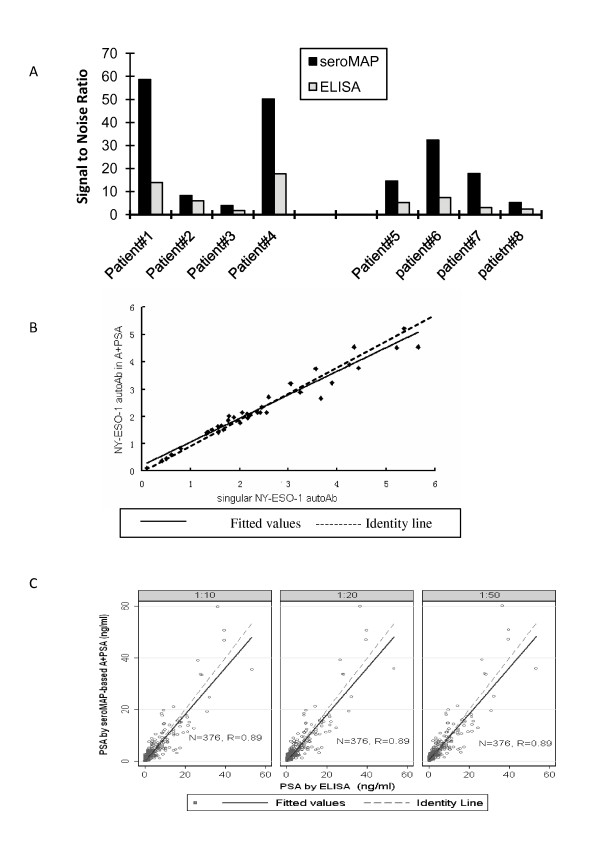
**Characteristics of the sero-MAP based multiplex assay measuring autoAb plus PSA.(A)**. seroMAP and ELISA were compared for measuring autoAb against the prototype NY-ESO-1:1-40 peptide and the XAGE-1b:1-25 peptide. Specific MFI ratios (or OD) from randomly selected seropositive patients were divided by the mean of 8 HD to represent signal-to-noise ratios of the seroMAP and ELISA approach. **(B)**. MFI ratios of autoAb against NY-ESO-1:1-40 versus a control obtained by the multiplex A+PSA and the singular assay had a correlation coefficient of 0.98 (n = 40), where the linear equation for NY-ESO-1 autoAb is A+PSA = 0.86*singular NY-ESO-1 + 0.20. **(C)**. Comparison of seroMAP-based A+PSA and classic ELISA for determining total PSA values (ng/ml) using serum samples of randomly selected 376 subjects. The three fitted linear regressions for 1:10, 1:20, and 1:50 dilution were A+PSA = 0.89*PSA+0.15, A+PSA = 0.90*PSA+0.30, and A+PSA = 0.90*PSA+0.43, respectively. Methods of determining PSA levels using seroMAP-based A+PSA and classic ELISA (American Qualex) were described in "Materials and Methods".

Following the identification and confirmation of peptide epitopes from the above-mentioned PCAA, each peptide was conjugated onto seroMAP microbeads with a specific region number (Materials and Methods). The ease of conjugating peptides over purified recombinant proteins onto seroMAP microspheres allowed multiplex detection of autoAb against the above-described peptide epitopes from XAGE-1b, SSX2,4, AMACR, p90 autoantigen, LEDGF, and NY-ESO-1 [18]. Specific MFI ratios, defined as the ratio of the MFI against a target peptide to the MFI against a control peptide, were compared with those from at least 8 HD, which was defined as the relevant signal-to-noise ratios for seroMAP-based approaches. Similarly, signal-to-noise ratios for ELISA-based approaches were determined (Materials and Methods). The seroMAP-based approach showed significantly improved signal-to-noise ratios over ELISA-based approach in measuring autoAb against a prototype NY-ESO-1:1-40 epitope among 4 randomly selected seropositive prostate cancer patients with 8 HD as controls (Figure 2A). Similarly, improved signal-to-noise ratios against the XAGE-1b epitope were observed using the seroMAP-based approach over ELISA.

### The multiplex A+PSA assay quantifies autoAb and total PSA in one reaction

To develop a multiplex assay that measures total PSA and autoAb in a single reaction, conventional PSA tests were first converted from ELISA to seroMAP-based approaches. PE-conjugated secondary Ab against human IgG and PSA were mixed in the multiplex assay to accommodate staining of autoAb and PSA binding to distinct seroMAP regions, allowing simultaneous quantification of autoAb plus PSA in one reaction (termed the A+PSA assay).

To ensure that the multiplex A+PSA assay did not interfere with the quantification of individual autoAb, autoAb against the prototype NY-ESO-1:1-40 epitope using the multiplex A+PSA assay were compared with those measured using seroMAP-based singular assays. It was found that autoAb against NY-ESO-1:1-40 measured by these two assays correlated markedly well among 40 randomly selected subjects (correlation coefficient was 0.98, Figure 2B). Similarly, purified PSA standards (n = 4) determined by the seroMAP-based A+PSA assay produced a trendline with a correlation coefficient of 0.98 with that obtained from a commercial ELISA kit (data not shown). For clinical samples, the correlation coefficient of PSA values obtained by ELISA (Figure 2C, x-axis) and the seroMAP-based A+PSA multiplex assay (y-axis) was 0.89 over a wide dynamic range from 0.1 to 60 ng/ml in 376 randomly selected subjects. Thus, the A+PSA assay format did not appear to produce interference by quantifying autoAb and PSA simultaneously in one reaction. In other words, the A+PSA assay is as specific as measuring individual autoAb and total PSA separately while providing the simplicity and cost-effectiveness of a multiplex assay that requires less sample and handling time of quantifying 6 or more autoAb and PSA simultaneously.

### The novel A+PSA index provides superior sensitivities and specificities over PSA alone in differentiating prostate cancer from non-malignant cases

**Figure 3:**
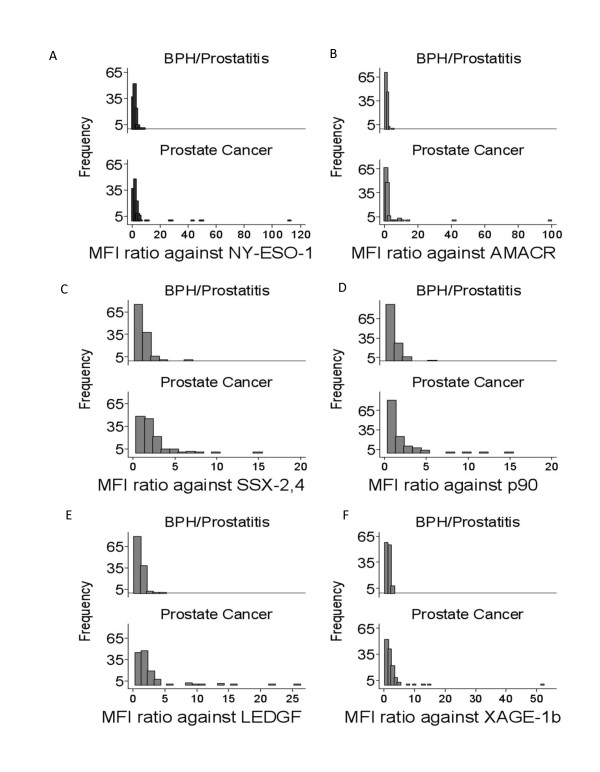
**Distribution of autoAb in patients with BPH/prostatitis and prostate cancer.** Histograms depicting the frequency or number of patients and their specific MFI ratios against NY-ESO-1:1-40 **(A)**, AMACR:341-371 **(B)**, SSX-2,4 **(C)**, p90 autoantigen **(D)**, LEDGF **(E)**, and XAGE-1b **(F) **in patients with BPH and/or prostatitis (n = 121) and prostate cancer (n = 131). Mean values of MFI ratios from 3 serum dilutions at 1/10, 1/20 and 1/50 were normalized against those obtained from HD (n = 124) to minimize experiment-to-experiment variations.

Pre-surgery serum samples from biopsy-confirmed prostate cancer patients (n = 131), BPH/prostatitis patients (n = 121) and healthy donors (n = 124), which were independent of the samples used in the epitope discovery phase, were subjected to determining total PSA and autoAb against the 6 defined PCAA epitopes. Histograms of the density or frequency against all 6 PCAA are depicted in Figure 3. Patients with non-malignant conditions had a narrower distribution of specific MFI ratios; meanwhile prostate cancer patients exhibited a much broader range of specific MFI ratios from 1 to nearly 300 for autoAb against NY-ESO-1. Histograms of other PCAA are shown in Figure 3B-F. The Kolmogorov-Smirnov tests for each of the 6 autoAb between prostate cancer and BPH/prostatitis groups were performed resulting in highly statistically significant differences between the two groups except for autoAb against AMACR. All four p-values of LEDGF, p90 autoantigen, SSX-2, 4 and XAGE-1b were less than 0.001 and the p-values of NY-ESO-1 and AMACR autoAb were 0.029 and 0.134, respectively.

**Figure 4:**
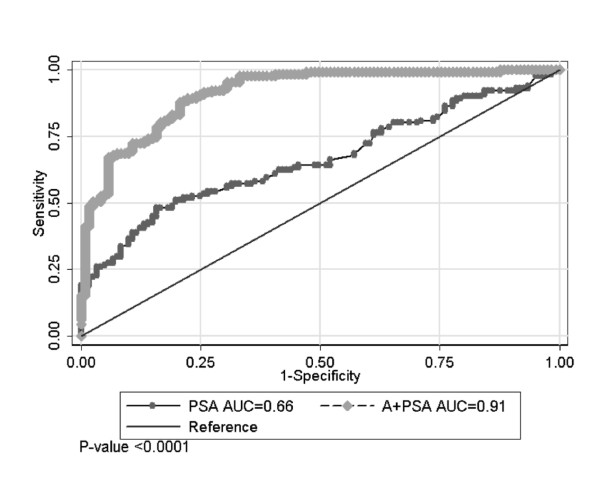
**An ROC curve comparing the A+PSA index and total PSA alone in differentiating the same group of prostate cancer and BPH/prostatitis patients as shown in Figure 3**. The distribution of total PSA values in samples used in this study are shown in Figure 1S.

A combined A+PSA index was created as the predicted probability of prostate cancer based on a logistic regression model. The classifications were made to prostate cancer if the probability was > = 0.5 and no cancer if the probability was <0.5. PSA alone and A+PSA at three different dilutions were compared with the mean and maximal dilution for sensitivity, specificity, accuracy and area under the curve (AUC). Mean values were selected as the optimal method to obtain sensitivity and specificity, and the receiver operating characteristic (ROC) curve was used to compare the diagnostic power between PSA alone and the combined A+PSA index for distinguishing prostate cancer from BPH and prostatitis. While the addition of any individual autoAb marker barely improved PSA test (data not shown), the addition of all 6 autoAb markers to PSA increased the assay sensitivity (success rate of predicting cancer), specificity (success rate of predicting non-cancer) and prediction accuracy (Table 2). AUC was also increased substantially from 0.66 for PSA alone to 0.91 for A+PSA. Figure 4 shows the ROC curves comparing the diagnostic power between PSA alone and the combined A+PSA index for distinguishing prostate cancer from nonmalignant BPH and/or prostatitis cases commonly seen in the clinic. The 95% bootstrap confidence interval in PSA alone was [0.59, 0.73], whereas the interval of A+PSA including the 6 autoAb was [0.88, 0.95]. A significant difference of AUC between A+PSA and PSA alone was observed (P < 0.001). This pilot study indicated potential benefits of the A+PSA assay in differentiating prostate cancer from non-malignant conditions commonly seen in the clinic.

**Table 2:** Comparison of A+PSA index and PSA based on mean values at 3 different dilutions

Variables	Sensitivity	Specificity	False positive	Accuracy	AUC
PSA alone in all patients	52% (68/131)	79% (95/121)	21% (26/121)	65%	0.66
A+PSA in all patients	79% (103/131)	84% (102/121)	16% (19/121)	81%	0.91 *P < 0.0001*

## Discussion

To improve PSA tests, a number of approaches have been investigated in the past, including isoforms of PSA such as free PSA and proPSA [22], new cancer biomarkers such as PCA3 [23], as well as combinatory assays measuring PSA and other parameters such as AMACR [24,25]. In this study, a novel assay platform was established that combines the quantification of autoAb against 6 TAA with a conventional biomarker, PSA in one reaction under the seroMAP platform. Even though the presence of autoAb against PSA reported in some patients [26] could in theory confound the quantification of PSA under the A+PSA assay platform, no significant discrepancy was observed among the more than 300 subjects. This assay platform employed B cell epitopes from previously defined PCAA and avoided peptides from out-of-frame and noncoding sequences, which have been observed in a large-scale autoAb signature study [4]. While the NY-ESO-1:1-40 peptide epitope has been validated by various investigators, the rest of the peptide epitopes panel is first reported in this study. Extensive validation is still necessary before moving forward into clinical trials. Serum samples from prostate cancer patients were collected at the time of diagnosis or surgery and thus included those with a wide range of Gleason scores. Since this pilot study was focused on developing a novel A+PSA platform and comparing A+PSA with the PSA assay, cohort size was not sufficient to match samples according to potential clinical co-founders. However, all samples themselves were handled and stored according to the same conditions prior to assay in order to minimize experiment-to-experiment variations.

It was predicted that peptide-based methods might lose conformational epitopes that could have been detected using full-length proteins. However, autoAb against NY-ESO-1 were detected in 7 of 131 or about 5% prostate cancer patients by seroMAP microspheres conjugated with a single peptide, higher than the reported frequency of 3.3% (3 out of 92) against the same peptide epitope using ELISA or 4.3% (4 out of 92) against the full-length NY-ESO-1 protein using ELISA in a previous study [18]. This result suggested that sero-MAP-based A+PSA assay against dominant peptide epitopes might compensate losses of conformational epitopes, and cover specific patient populations otherwise overlooked using ELISA methods coated with full-length proteins. Considering that sero-MAP based A+PSA assay is performed entirely in liquid phase with enhanced kinetics over surface-bound ELISA, we will investigate whether compensation for conformational epitopes by A+PSA assay also occurs for other PCAA. Furthermore, the multiplex A+PSA assay requires less than 20 μl serum samples for three different dilutions altogether, much fewer handling steps to be completed within two and half hours, making it user-friendly to clinical laboratories.

In order to deliver a fully functional A+PSA assay to clinical laboratories, we plan to cross-validate with larger and broader patient cohorts including sex, age, and racial/ethnic background matched HD and non-cancer controls. In this pilot study, the A+PSA index also reduced the false positive rate of PSA tests, which suggested its potential implications in aiding in prostate cancer diagnosis. Thus, patients with lung cancer and colon cancer, two common cancers for elder men will also be included in the cross-validation in order to enhance the prostate cancer specificity of the assay. Once an optimized A+PSA assay has been developed, prospective studies comparing A+PSA with PSA alone as well as emerging genotype-based tests such as urine PCA3/PSA mRNA ratio detection and TMPRSS2-ERG fusion gene [27], will be conducted. Other areas of potential implication such as differentiating lethal from indolent prostate cancer will also be investigated in the future. The versatile nature of the multiplex A+PSA assay allows the addition and deletion of specific peptide epitopes to the panel in order to define correlations with the intended clinical implications.

## Conclusions

The A+PSA assay represents the first multiplex assay that integrates autoAb signatures with a conventional cancer biomarker PSA in a single reaction. Designed to be user-friendly to clinical laboratories, the A+PSA assay has the potential to aid in the diagnosis and prognosis of prostate cancer.

## List of Abbreviations

TAA: tumor-associated antigen; PSA: prostate specific antigen; HD: healthy donors; NSCLC: non-small cell lung cancer; A+PSA: autoantibody plus PSA; BPH: benign prostatic hyperplasia; Ab: antibody; PCAA: prostate cancer-associated antigen; OD: optical density; MFI: mean fluorescent intensity; ROC: receiver operating characteristic.

## Competing interests

The authors declare that they have no competing interests.

## Authors' contributions

CX carried out the serological assays, participated in the data analysis and drafted the results of the manuscript. HK and GL participated in the design of the study and carried out the statistical analysis. JH and AK participated in the identification of peptide epitopes. BG helped with the seroMAP-based assays. JR, DC, AP participated in the overall design of the study and interpretation of results. ML conjugated all peptides/proteins to microspheres. RP, LS, AT, GW, and HM collected patients' samples and provided their clinical information. GZ conceived of the study, participated in its design and coordination, and drafted the manuscript. All authors read and approved the final manuscript.

## Supplementary Material

Additional file 1 **Total PSA values are shown for the HD (n = 124), BPH/prostatitis (n = 121) and prostate cancer patients (n = 131) involved in the comparison of A+PSA and PSA alone**. There are 1 and 28 patients with PSA equal or above 15 ng/ml (filled triangles) in the BPH/prostatitis and prostate cancer group, respectively. Click here for file

Additional file 2 **Verification of peptide epitopes by Western blot**. Western blots against 50 ng of purified recombinant C-terminal portion of LEDGF protein (amino acid 322-530, Abcam Biotechnology, Cambridge, MA) in lane 3 **(A) **and AMACR protein (Abcam Biotechnology) in lane 3 **(B)**. In both cases, 10 and 20 μg of 293 cell lysates were compared as controls (lanes 1 and 2 of each panel). Serum samples from prostate cancer patients with LEDGF and AMACR specific autoAb based on peptide screening were used at 1 to 500 dilutions for the blot. Molecular weight standards (kDa) are shown on the sides. **(C)**. Western blot against bacterial lysate expressing recombinant SSX-2,4, the C-terminal half of p90 autoantigen, and NY-ESO-1 (lane 1, 2, and 3 respectively in each panel). The left panel was blotted with Ab against the polyhistidine tag to locate protein bands corresponding to SSX-2,4, p90, and NY-ESO-1 (as a positive control). The center and right panel were blotted with serum samples from prostate cancer patients with positive reactions against p90 and SSX2,4 peptides (p90 and SSX2,4 proteins are circled), respectively. Click here for file
