# Supplementary material for: A novel multiplex assay combining autoantibodies plus PSA has potential implications for classification of prostate cancer from non-malignant cases
Source: J Transl Med. 2011 Apr 19;9:43. doi: 10.1186/1479-5876-9-43 (PMC3102624; doi:10.1186/1479-5876-9-43)
Supplement: Additional file 1 — Total PSA values are shown for the HD (n = 124), BPH/prostatitis (n = 121) and prostate cancer patients (n = 131) involved in the comparison of A+PSA and PSA alone. There are 1 and 28 patients with PSA equal or above 15 ng/ml (filled triangles) in the BPH/prostatitis and prostate cancer group, respectively. [file 1479-5876-9-43-S1.DOC]

Additional Files

**
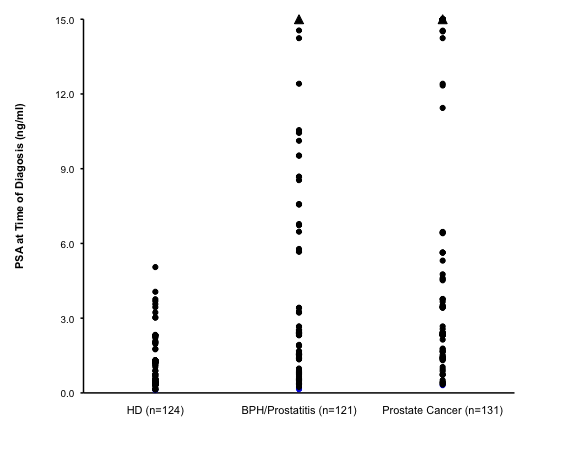
Additional file 1:**

Total PSA values are shown for the HD (n=124), BPH/prostatitis (n=121) and prostate cancer patients (n=131) involved in the comparison of A+PSA and PSA alone. There are 1 and 28 patients with PSA equal or above 15 ng/ml (filled triangles) in the BPH/prostatitis and prostate cancer group, respectively.
