# Supplementary material for: A novel multiplex assay combining autoantibodies plus PSA has potential implications for classification of prostate cancer from non-malignant cases
Source: J Transl Med. 2011 Apr 19;9:43. doi: 10.1186/1479-5876-9-43 (PMC3102624; doi:10.1186/1479-5876-9-43)
Supplement: Additional file 2 — Verification of peptide epitopes by Western blot. Western blots against 50 ng of purified recombinant C-terminal portion of LEDGF protein (amino acid 322-530, Abcam Biotechnology, Cambridge, MA) in lane 3 (A) and AMACR protein (Abcam Biotechnology) in lane 3 (B). In both cases, 10 and 20 μg of 293 cell lysates were compared as controls (lanes 1 and 2 of each panel). Serum samples from prostate cancer patients with LEDGF and AMACR specific autoAb based on peptide screening were used at 1 to 500 dilutions for the blot. Molecular weight standards (kDa) are shown on the sides. (C). Western blot against bacterial lysate expressing recombinant SSX-2,4, the C-terminal half of p90 autoantigen, and NY-ESO-1 (lane 1, 2, and 3 respectively in each panel). The left panel was blotted with Ab against the polyhistidine tag to locate protein bands corresponding to SSX-2,4, p90, and NY-ESO-1 (as a positive control). The center and right panel were blotted with serum samples from prostate cancer patients with positive reactions against p90 and SSX2,4 peptides (p90 and SSX2,4 proteins are circled), respectively. [file 1479-5876-9-43-S2.DOC]

**Additional file 2:**


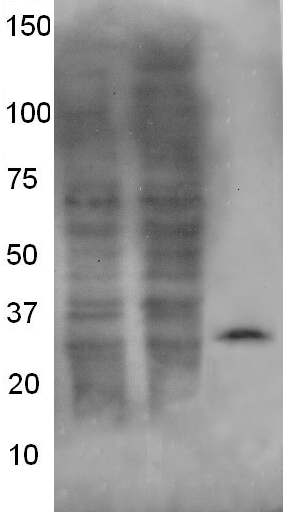

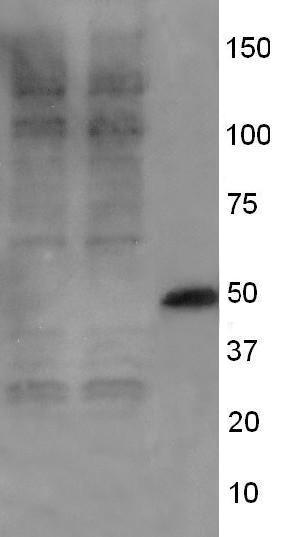


1 2 3 1 2 3

A.

B.

C.

MMW 1 2 3 1 2 3 1 2 3

MW 1 2 3 1 2 3 1 2 3

64 kDa

49 kDa

37 kDa

26 kDa


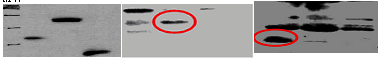


Western blots against 50 ng of purified recombinant C-terminal portion of LEDGF protein (amino acid 322-530, Abcam Biotechnology, Cambridge, MA) in lane 3 **(A)** and AMACR protein (Abcam Biotechnology) in lane 3 **(B)**. In both cases, 10 and 20 μg of 293 cell lysates were compared as controls (lanes 1 and 2 of each panel). Serum samples from prostate cancer patients with LEDGF and AMACR specific autoAb based on peptide screening were used at 1 to 500 dilutions for the blot. Molecular weight standards (kDa) are shown on the sides. **(C).** Western blot against bacterial lysates expressing recombinant SSX-2,4, the C-terminal half of p90 autoantigen, and NY-ESO-1 (lane 1, 2, and 3 respectively in each panel). The left panel was blotted with Ab against the polyhistidine tag to locate protein bands corresponding to SSX-2,4, p90, and NY-ESO-1 (as a positive control). The center and right panel were blotted with serum samples from prostate cancer patients with positive reactions against p90 and SSX2,4 peptides (p90 and SSX2,4 proteins are circled), respectively.

664 kDa

49 kDa

37 kDa

26 kDa
